# Infective endocarditis requiring ICU admission: epidemiology and prognosis

**DOI:** 10.1186/s13613-015-0091-7

**Published:** 2015-12-01

**Authors:** Olivier Leroy, Hugues Georges, Patrick Devos, Steve Bitton, Nathalie De Sa, Céline Dedrie, Sébastien Beague, Pierre Ducq, Claire Boulle-Geronimi, Damien Thellier, Fabienne Saulnier, Sebastien Preau

**Affiliations:** Service de Réanimation Médicale et Maladies Infectieuses, Hôpital Chatiliez, 135 rue du Président Coty, Tourcoing, 59200 France; Département de bio statistique, CHU de Lille, 59037 Lille Cedex, France; Pôle de Réanimation, Hôpital R. Salengro, CHU de Lille, Avenue du Professeur E. Laine, 59037 Lille Cedex, France; Service de Réanimation Polyvalente, Centre Hospitalier Jean Bernard, Avenue Désandrouin, 59322 Valenciennes Cedex, France; Service de Réanimation Polyvalente, Hôpital Victor Provost, Rue de Barbieux, 59056 Roubaix Cedex, France; Service de Réanimation Polyvalente, Centre Hospitalier de Dunkerque, Avenue Louis Herbeaux, 59385 Dunkirk, France; Service de Réanimation Polyvalente, Centre Hospitalier de Boulogne-sur-Mer, Allée Jacques Monod, 62321 Boulogne-Sur-Mer Cedex, France; Service de Réanimation Polyvalente, Centre Hospitalier de Douai, Route de Cambrai, 59507 Douai Cedex, France

**Keywords:** Critical care medicine, Infective endocarditis, Cardiac surgery, Infectious diseases, Prognostic

## Abstract

**Background:**

Very few studies focused on patients with severe infective endocarditis (IE) and multiple complications leading to Intensive Care Unit (ICU) admission. Studied primary outcomes depended on the series and multiple prognostic factors have been identified. Our goal was to determinate characteristics of patients, in-hospital mortality and independent prognostic factors in an overall population of patients admitted to ICU for a left-sided, definite, active and severe IE.

**Methods:**

Retrospective study performed in 9 ICUs during an 11-year period.

**Results:**

Data of 248 patients (mean age = 62.4 ± 13.3 years; 63.7 % male) were studied. Native and prosthetic valves were involved in 195 and 53 patients, respectively. Causative pathogens, identified in 225 patients, were mainly streptococci (45.6 %) and staphylococci (43.4 %). On ICU admission, 127 patients exhibited extra-cardiac involvement. Ninety-five patients had one or more neurological complications, as followed: ischemic stroke (*n* = 66), cerebral hemorrhage (*n* = 31), meningitis (*n* = 16), brain abscess (*n* = 16), and intracranial mycotic aneurysm (*n* = 10). Criteria prompting to cardiac surgery appeared during ICU stay for 186 patients and between ICU and hospital discharges in 5 patients. Due to contra-indications, surgery required by IE was only performed during hospitalization in 125 patients. Moreover, surgery was considered adequate according to usual guidelines in 76 of 191 patients with indication(s) of valvular surgery: for patients with surgical procedure considered as emergency (*n* = 69), 17 surgical procedures underwent within the first 24 h following indication; for patients with urgent surgical indication (*n* = 102), surgery was performed during the first week following indication in 40 patients; finally, elective surgery (*n* = 20) was performed for 19 patients. During hospitalization, 103 (41.5 %) patients died. Four independent prognostic factors were identified: SAPS II > 35 (AOR = 2.604; 95 % CI: 1.320–5.136; *p* = 0.0058), SOFA > 8 (AOR = 3.327; 95 % CI: 1.697–6.521; *p* = 0.0005), IE due to methicillin resistant *Staphylococcus aureus* (AOR = 4.981; 95 %CI = 1.433–17.306; *p* = 0.0115) and native IE (AOR = 0.345; 95 % CI: 0.169–0.703; *p* = 0.0034).

**Conclusions:**

Mortality in patients admitted to ICU for left-sided IE remains high, especially in cases of endocarditis due to methicillin resistant *Staphylococcus aureus*, when organ failures occur and ICU scores are high.

## Background

The annual incidence of infective endocarditis (IE) in France at the beginning of the 21st century is around 33 cases per million inhabitants [1]. Despite advances in diagnosis and medico-surgical treatment, the in-hospital mortality rate remains high, since ranging from 15 to 22 % [1–3]. Most recent data underline that now *Staphylococcus aureus* is the most common cause of IE and that approximately 50 % of patients underwent early valve replacement or repair [2, 3].

Many complications can arise during the evolution of IE. Some are inaugural, the others come as the diagnosis has already been raised. Most of them can justify the admission of the patient in intensive care unit (ICU). Unfortunately, very few studies focused on patients with severe IE and multiple complications leading to ICU admission [4–7]. Therefore, we conducted a multicenter retrospective analysis of all consecutive critical left-sided IE patients to determinate characteristics of patients, in-hospital mortality and independent prognostic factors in the overall population.

## Patients and methods

### Study design and patients

In 9 ICUs of 7 hospitals (Boulogne sur Mer, Douai, Dunkerque, Valenciennes, Lille, Roubaix, and Tourcoing) in Nord-Pas de Calais, an area from North of France, the charts of all consecutive patients admitted to the ICU with a diagnosis of IE between January 2002 and December 2012 were reviewed (OL, HG, SBi, ND, CD, SBe, PDu, CBG and SP). Information collected from medical records was anonymously entered into a database and reviewed for data entry errors and/or inconsistencies (OL, HG, SBi and SP). In agreement with French regulations concerning observational studies that do not modify existing diagnosis or therapeutic strategies, no ethics committee approval was required to conduct the study.

Adult patients were enrolled in the study if they had a left-sided, definite, active and severe IE requiring ICU admission. IE involving mitral and/or aortic valves was defined as left-sided. Patients with both left- and right-sided IE and left-sided IE associated with infection of a cardiac implantable electronic device (CIED) were considered as left-sided IE. Definite IE was defined according to modified Duke criteria [8]. Endocarditis was defined as active if the patient was admitted in ICU before or within the first 30 days of antimicrobial treatment. IE was considered as severe when associated with any of the following criteria: acute respiratory failure requiring mechanical ventilation, shock, Simplified Acute Physiology Score (SAPS) II ≥20 and Sepsis-related Organ Failure Assessment (SOFA) score ≥3.

Patients with right-sided IE or infection of a cardiac implantable electronic device without left valves involvement were not included as well as patients with IE acquired during ICU stay, or admitted in ICU after valve surgery for IE and, finally, those with possible IE.

### Data collection and definitions

For each patient, IE was diagnosed when required modified Duke criteria were obtained. As all these criteria could not be present on ICU admission, there may be a delay between ICU admission and IE diagnosis that was determined. When IE was diagnosed, we collected for each patient information about demographics, pre-existing comorbidities, condition at ICU admission, initial valve status, origin of infection, microbiological data, echocardiographic data, and extra-cardiac involvement revealed by initial work-up. As this study was retrospective, the initial work-up was not standardized and radiological examinations were performed at the discretion of physicians. During the ICU stay, the antimicrobial treatment, the occurrence of complications, indications for surgery, timing of surgical procedure were recorded. Finally, outcome was evaluated.

Underlying conditions were evaluated by the Charlson score [9]. Severity of illness at ICU admission was assessed by the SAPS II, and SOFA score [10, 11]. Prosthetic valve IE was defined as infection occurring on any type of tissue or mechanical device. Hospital-acquired IE was defined as infection occurring more than 72 h after admission to the hospital or acquired in association with a significant invasive procedure performed during a recent hospitalization within 8 weeks of this hospitalization [12]. Microbiological diagnosis was assessed according to modified Duke criteria. Echocardiographic data recorded were regurgitant valve, vegetation length and location. Follow-up examinations were performed to monitor vegetation size and to detect the occurrence of complications. Antimicrobial therapy was considered adequate if it included antibiotic(s) usually proposed by current guidelines [13]. IE complications on ICU admission such as congestive heart failure, septic shock, and extra-cardiac involvement mainly due to systemic embolic events were defined according to current guidelines [13]. For neurological involvement, five complications were distinguished according to the results of brain imaging (magnetic resonance imaging and/or computerized tomography scanning) and cerebrospinal fluid analyses performed on initial work-up: ischemic stroke, cerebral hemorrhage, meningitis or meningeal reaction, brain abscess, and intracranial mycotic aneurysm.

Indications (heart failure, prevention of embolic events and uncontrolled infection) and timing of surgery (emergency, urgent and elective) were defined by current guidelines [13]. Surgery was considered adequate when surgical procedure was performed accordingly to such guidelines. In-hospital mortality was defined as death occurring within the same hospitalization as ICU admission, regardless of its cause.

### Statistical analysis

Descriptive analyses were performed to check and summarize the data. Quantitative variables are reported as mean ± standard deviation. Qualitative variables are reported as number and percentage. Continuous variables were compared using the Student’s t test. Categorical variables were compared using the Chi-square test or Fisher’s exact test when Chi square was not appropriate. Differences between groups were considered to be significant for variables yielding a *p* value ≤0.05. A stepwise logistic regression analysis was performed to identify risk factors associated with in-hospital mortality, regardless of its cause. In order to identify independent risk factors for mortality, variables were included in the multivariate model if the *p* value was ≤0.05 in bivariate analysis. Adjusted odds ratios (AOR) were computed using logistic regression analysis including the independent predictors of mortality.

All statistical analyses were performed using SAS software, V9.1.

## Results

During the study period, 352 patients with IE were admitted in one of the 9 ICUs of our group. We excluded 104 patients for the following reasons: right-sided IE or infection of a cardiac implantable electronic device without left valves involvement (*n* = 41); diagnosis of IE non definite (*n* = 34); non severe IE (*n* = 24); IE acquired during ICU stay (*n* = 5). Finally, 248 patients with severe, active, definite and left-sided IE were studied. Among them, two IE major criteria were present in 232 patients and one major criterion with 2 or 3 minor criteria was present in the remaining 16 patients. One hundred forty-eight (59.7 %) patients were admitted in a tertiary care hospital with a cardiac surgery department (Lille) and 100 (40.3 %) were admitted in a general hospital without any cardiac surgery department. The mean delay between ICU admission and IE diagnosis was 1.75 ± 3.74 days. Main patients’ characteristics on ICU admission and on IE diagnosis are summarized in Table 1.

**Table 1:** Main patients’ characteristics on ICU admission and on IE diagnosis

Patients’ characteristics	
Age (years)	62.4 ± 13.3
Sex: M/F	158/90
Charlson score	4.58 ± 2.70
SAPS II	36.7 ± 16.7
SOFA score	7.0 ± 3.7
Main indications for ICU admission	
Septic shock	54 (21.8)
Severe valvular regurgitation	46 (18.55)
Cardiogenic shock	39 (15.7)
Acute renal failure	36 (14.5)
Acute respiratory failure requiring mechanical ventilation	28 (11.3)
Neurological complications	17 (6.85)
Community-acquired/hospital-acquired IE	215 (86.7)/33 (13.3)
Native valve/prosthetic valve IE	195 (78.6)/53 (21.4)
Valve involvement	
Aortic	156
Mitral	152
Tricuspid	9
Pulmonary	1
Multiple valve involvement	66
Aortic plus mitral valves	53
Aortic plus tricuspid valves	3
Aortic plus pulmonary valves	1
Aortic plus mitral plus tricuspid valves plus CIED	1
Aortic plus mitral valves plus CIED	3
Aortic valve plus CIED	1
Mitral plus tricuspid valves	3
Mitral plus tricuspid valves plus CIED	1
Portal of entry of IE	126 (50.8)
Skin or soft tissue	36
Dental	24
Upper respiratory tract	9
Genitourinary tract	13
Digestive tract	23
Intra-venous drug abuse	2
Cardiovascular procedure or vascular access	19

The results are given as *n* (%) or median ± SD

When indicated *SAPS II* Simplified Acute Physiologic Score II, *SOFA* sepsis-related organ failure assessment score, *CIED* cardiac implantable electronic device

Echocardiographic examinations revealed mitral vegetation(s) in 137 patients. Vegetations were large (>10 mm) in 67 patients and very large (>15 mm) in 41 patients. Mitral regurgitation was severe (3 to 4+) in 56 patients. One hundred and twenty-three patients exhibited aortic vegetation(s). They were large and very large in 51 and 24 patients, respectively. Aortic regurgitation was severe in 66 patients. An annular abscess was observed in 67 patients. Six patients had a pericardial effusion. Finally, the mean value of left ventricular ejection fraction was 55.0 ± 11.6 %. It was <35 % in 16 patients, >35 % and <50 % in 82, >50 % in 144, and finally not determined in 6.

Causative pathogen was identified in 225 (90.7 %) patients. Blood and leaflet cultures were positive in 222 and 25 cases, respectively. Two hundred and thirty-five causative pathogens were identified. IE was polymicrobial in 9 patients. The most common pathogens were streptococci (45.6 %), and staphylococci (43.4 %) (Table 2).

**Table 2:** Causative microorganisms (*n* = 235) isolated from cases of active IE

Micro organism	*N* (%)
*Streptococcus* spp.	107 (45.6)
Beta-haemolytic *Streptococcus* (groups A, B, C and G)	27
Oral *Streptococcus*	24
*Enterococcus* spp.	24
Group D *Streptococcus*	19
*Streptococcus pneumoniae*	13
*Staphylococcus aureus*	90 (38.3)
Methicillin-susceptible	74
Methicillin-resistant	16
Coagulase-negative *Staphylococcus*	12 (5.1)
Gram-negative bacilli	14 (6.0)
*Haemophilus influenzae*	2
*Enterobacteriaceae*	11
*Pasteurella multocida*	1
Gram positive bacilli	1 (0.4)
*Candida* spp. and *Aspergillus* spp.	4 (1.7)
Other	7 (3.05)

On ICU admission and during the initial course of IE, 127 patients (51.2 %) exhibited extra-cardiac involvement. Main involved organ was the central nervous system. According to neurological investigations performed on 184 patients (74.2 %) (computerized tomography scanning *n* = 160, magnetic resonance imaging, and cerebrospinal fluid analyses *n* = 25), 139 neurological complications were found in 95 patients, as followed: ischemic stroke (*n* = 66), cerebral hemorrhage (*n* = 31), meningitis (*n* = 16), brain abscess (*n* = 16), and intracranial mycotic aneurysm (*n* = 10). Moreover, according to chest (*n* = 105), bone (*n* = 75) and abdominal (*n* = 144) computerized tomography scans, abdominal ultrasonography (*n* = 34), lung (*n* = 8) and bone (*n* = 13) scans, and bone magnetic resonance imaging (*n* = 24), systemic embolic or metastatic infective events involving spleen (*n* = 33), bone and joints (*n* = 22), kidneys (*n* = 21), lung (*n* = 3) and liver (*n* = 3) were found.

All patients received an antimicrobial treatment. This treatment was adequate in 206 (83.1 %) patients. During ICU stay, surgery was indicated for 186 (75 %) patients. The timing of surgical procedure was considered as emergency in 69 (37.1 %) patients, urgent in 102 (54.9 %) patients and elective in 15 (8 %) patients. Main indications and contra-indications for surgery are summarized in Table 3. Surgery was performed in 99 patients during the ICU stay (Table 4). Among the 101 patients without contra-indications to surgery, 84 (83.2 %) patients underwent surgery during ICU stay. Among the 85 patients with contra-indications, surgery was nonetheless performed in 15 (17.6 %) patients. In these latter patients, contra-indications were hemorrhagic stroke (*n* = 10), multiple organ failure (*n* = 3) and severe underlying diseases (*n* = 2). It could be noticed that only two of them, exhibiting multiple organ failure, died. Moreover, 21 patients with surgery indicated during ICU stay and 5 patients with ultimately appeared indication underwent surgical procedure between ICU and hospital discharges. Thus, during hospitalization, a surgical procedure was required by IE in 191 patients and was performed for 125 patients. Surgical procedure was required more often for patients admitted in a tertiary care hospital with a cardiac surgery department (*n* = 127/148) than for admitted in a general hospital without any cardiac surgery department (*n* = 64/100) (*p* < 0.001).

**Table 3:** Main Indications and contra-indications for valvular surgery during ICU stay

Timing of surgery	Indications	Number of patients with contra-indications	Contra-indications
Emergency *n* = 69	Cardiogenic shock *n* = 41 Refractory pulmonary oedema *n* = 28	33 (47.8 %)	Multiple organ failure *n* = 16 Hemorrhagic stroke *n* = 8 Severe underlying diseases *n* = 7 Risk of extra cerebral hemorrhage *n* = 2
Urgent *n* = 102	Very large vegetations = 43 Large vegetations and embolic episodes *n* = 21 Annular abscess n = 27 Uncontrolled infection *n* = 8 Severe acute regurgitation *n* = 3	50 (49 %)	Risk of cerebral hemorrhage *n* = 22 Multiple organ failure *n* = 16 Severe underlying diseases *n* = 11 Risk of extra cerebral haemorrhage *n* = 1
Elective *n* = 15	Severe regurgitation without heart failure *n* = 12 Severe prosthetic dehiscence *n* = 3	2 (13.3 %)	Severe underlying diseases *n* = 1 Multiple organ failure *n* = 1

**Table 4:** In hospital mortality according to surgery during ICU stay and in-hospital mortality

Timing of surgery	Number of patients	Number of patients with surgery performed during ICU stay	In hospital mortality *n* (%)	Number of patients with surgery not performed during ICU stay	In hospital mortality *n* (%)
Patients with contra-indications to surgery *n* = 85					
Emergency	33	6	1 (16.7 %)	27	25 (92.6 %)
Urgent	50	8	1 (12.5 %)	42	30 (71.4 %)
Elective	2	1	0	1	0
Patients without contra-indications to surgery *n* = 101					
Emergency	36	36	6 (16.6 %)	0	0
Urgent	52	41	11 (21.2 %)	11	0
Elective	13	7	0	6	2 (33.3 %)

For patients with surgical procedure considered as emergency (*n* = 69), 17 surgical procedure underwent within the first 24 h following indication. For patients with urgent surgical indication (*n* = 102), surgery was performed during the first week following indication in 40 patients. Finally elective surgery (*n* = 20) was performed for 19 patients. So, surgery was considered adequate according to usual guidelines in 76 of 191 (39.8 %) patients with indication(s) of valvular surgery. Surgery was more often adequate for patients admitted in a tertiary care hospital with a cardiac surgery department (*n* = 57/127) than for patients in a general hospital without any cardiac surgery department (*n* = 19/64) (*p* < 0.001).

During hospitalization, 103 (41.5 %) patients died. Main significant factors associated with in-hospital mortality in bivariate analysis are reported in Table 5. Factors assessing severity of underlying diseases and/or IE on ICU admission were associated with a significant increased mortality as well as prosthetic and staphylococcal IE. Conversely, native IE, IE due to *Streptococcus* and therapeutic measures such as surgery during ICU stay, adequate surgery and adequate antimicrobial treatment were associated with a significant decreased mortality. Multivariate analysis including all significant variables in bivariate analysis (*p* < 0.05), except surgery during ICU stay, surgery (overall) and adequate surgery, identified 4 independent prognostic factors. They were SAPS II > 35 (AOR = 2.604; 95 % CI: 1.320–5.136; *p* = 0.0058), SOFA > 8 (AOR = 3.327; 95 % CI: 1.697–6.521; *p* = 0.0005), IE due to methicillin resistant *Staphylococcus aureus* (AOR = 4.981; 95 %CI = 1.433–17.306; *p* = 0.0115) and native valve IE (AOR = 0.345; 95 % CI: 0.169–0.703; *p* = 0.0034).

**Table 5:** Bivariate analysis of risk factors for in-hospital mortality

Factor	Survivors *n* = 145	Non-survivors *n* = 103	*p*
Charlson score	4.18 ± 2.80	5.15 ± 2.46	0.003
SAPS II	30.99 ± 13.16	44.60 ± 17.95	<0.0001
SAPS II > 35	43 (38.4 %)	69 (61.6 %)	<0.0001
SOFA score	5.65 ± 2.77	8.93 ± 3.91	<0.0001
SOFA score >8	30 (33 %)	61 (67 %)	<0.0001
Glasgow Coma Score <9	7 (17.9 %)	32 (82.1 %)	<0.0001
ICU admission for septic shock	17 (31.5 %)	37 (68.5 %)	<0.0001
ICU admission for cardiogenic shock	16 (41 %)	23 (59 %)	0.016
ICU admission for acute respiratory failure	22 (78.6 %)	6 (21.4 %)	0.02
ICU admission for severe valvular regurgitation	40 (87 %)	6 (13 %)	<0.0001
Mitral IE	80 (52.6 %)	72 (47.4 %)	0.02
Native IE	121 (62.05 %)	74 (37.95 %)	0.03
Prosthetic IE	24 (45.3 %)	29 (54.7 %)	0.03
Annular abscess	31 (46.3 %)	36 (53.7 %)	0.02
Severe aortic regurgitation	50 (75.8 %)	16 (24.2 %)	0.0009
Left ventricular ejection fraction (%)	57 ± 11	52 ± 13	0.005
IE due to *Streptococcus* spp.	72 (67.3 %)	35 (32.7 %)	0.01
IE due to MSSA	36 (48.70 %)	38 (51.3 %)	0.04
IE due to MRSA	4 (25 %)	12 (75 %)	0.005
Adequate antimicrobial treatment	127 (61.7 %)	79 (38.3 %)	0.02
Surgery during ICU stay	80 (80.8 %)	19 (19.2 %)	<0.0001
Surgery (overall)	102 (81.6 %)	23 (18.4 %)	<0.0001
Adequate surgery	65 (85.5 %)	11 (14.5 %)	<0.0001

*MSSA* methicillin-susceptible *Staphylococcus aureus*, *MRSA* methicillin-resistant *Staphylococcus aureus*

To focus on the 16 patients with IE due to methicillin resistant *Staphylococcus aureus*, we could add that 12 had a native valve IE. Infection was hospital-acquired in 8 cases. Main portal of entry were skin or soft tissue (*n* = 4) and vascular access infections (*n* = 4). On ICU admission, the mean SAPS II was 44.6 ± 23.7 and 7 patients exhibited septic shock. Annular abscess was observed in 7 patients and neurological complications occurred in 7 patients. Antimicrobial treatment was considered inadequate in 4 patients (vancomycin without gentamicin in 2 cases of native valve IE and vancomycin + gentamicin without rifampin in 2 cases of prosthetic valve IE). Surgery was indicated for 11 patients (emergency *n* = 3; urgent *n* = 7; elective *n* = 1) but was adequate in only 3 patients. During hospitalization, 12 patients died (in ICU, *n* = 10).

## Discussion

We report the results of a retrospective multicenter study on 248 patients with severe, active, definite and left-sided IE requiring ICU admission. Main causative pathogens are equally represented by streptococci and staphylococci. During ICU stay, surgery was indicated for 75 % of patients but only 53 % of them underwent surgical procedures during ICU stay. Overall in-hospital mortality was 41.5 %. Independent factors associated with prognosis were SAPS II on ICU admission >35, SOFA on ICU admission >8, IE due to methicillin resistant *Staphylococcus aureus* and native IE.

Current data suggest that staphylococci are the most common causative pathogens of IE. In the overall population of adults with definite IE admitted to hospital, *S. aureus* accounted for 26.6–36.2 % of causal agents [2, 14]. In series including only adult patients admitted to ICU with infective endocarditis, *S. aureus* represented 45–56 % of identified causative organisms [4, 7]. Our results could appear surprising since even if staphylococci are involved in 43.4 % of patients, they are less frequent causative organisms than streptococci involved in 45.6 % of patients. However, they are similar to those reported more than 10 years ago by Hoen et al. in France and Hasbun et al. in USA [15, 16]. In these series having included patients in 1999 and between 1990 and 2000, streptococci are involved in 48 and 58 %, respectively. We have no clear explanation about these microbiological differences between our study and those reporting data from patients admitted in ICU [4, 7]. Nevertheless, studied patients could be a little different. In the study from Mourvillier and colleagues, prosthetic valve IE are more frequent than in our series (40.6 vs. 21.4 %) and, if we focused only on native IE, streptococci and staphylococci are equally involved as causative organisms [4]. In the study reported by Samol and colleagues, 31 % of patients had right-sided endocarditis and it is well known that *S. aureus* is then the most common pathogen [7, 17].

In our study, a surgical procedure required by IE was performed during hospitalization for 125 patients (50.4 %). In series focusing on patients admitted to ICU for IE, 35–52 % of patients underwent surgery [4, 6, 7]. Rather than these gross percentages, an important point is, in our mind, the percentage of performed surgical procedures among patients for which indications for surgery emerge during ICU stay. Literature data are unfortunately scarce. In our series, surgery was indicated for 186 (75 %) patients but only 99 (53 %) of them underwent surgical procedures during ICU stay. In the study reported by Mirabel et al., the percentage appears higher since 100 of 158 patients with recommended surgical procedure underwent surgery [6]. Unfortunately, in this series, the timing of surgical procedure (emergency, urgent and elective) was only reported for patients undergoing surgery. So, it was not possible to determine the adequacy of surgery according to the timing, and consequently, to compare these results with ours. It’s a shame because it would have been interesting to known if the low percentage of adequate surgery observed in our series when timing was considered as emergency or urgent was also observed in other studies. In our series, it could be also noticed that among the 85 patients with contra-indications to surgery, 15 underwent nonetheless surgery and that 13 of them survived. Finally, surgery was more often adequate for patients admitted in a tertiary care hospital with a cardiac surgery department than for patients in a general hospital without any cardiac surgery department. Such a result reinforces recent recommendations for referring complicated IE patients to tertiary care hospitals in which a collaborative approach of IE involving notably a cardiac surgeon is possible [18].

The impact of surgery on IE prognosis was the subject of numerous studies. Despite some conflicting results, surgical therapy appears most often associated with an improved early and late survival both in the overall population of patients than in patients admitted to ICU [4, 6, 7, 19–25]. In our series, in-hospital mortality was 41.5 %. In similar series, mortality rates varied between 30 and 45 %, and apart surgery, identified independent prognostic factors were septic shock, cerebral emboli, immunosuppression, neurological failure, severe comorbidities, *S. aureus* IE and SAPS II [4, 5, 7]. Most of these factors appear in our series as significant prognostic factors in bi variate analysis. Among them, we could notice that IE due to *Streptococcus* spp. were associated with a lower mortality than IE due to *Staphylococcus* spp. and that adequacy of antimicrobial and of surgical treatment also appeared as factors associated with survival. However, in our study, we willingly chose to not enter in multivariate analysis the significant factors about surgery identified in bivariate analysis (surgery during ICU stay, surgery and adequate surgery) since the overall population was not affected by these prognostic factors. Our multivariate analysis identified 4 independent factors. They were SAPS II > 35 (AOR = 2.604), SOFA > 8 (AOR = 3.327), IE due to methicillin resistant *Staphylococcus aureus* (AOR = 4.981) and native IE (AOR = 0.345). The fact that scores assessing severity and/or organ failure on ICU admission are independent prognostic factors is not surprising since they are usually found in all studies focusing on prognostic of ICU patients. The protective role of the native character of endocarditis is not, in our opinion, surprising since the deleterious role of the prosthetic character of endocarditis is well known [3]. In example, in the study reported by Murdoch et al. including 2781 patients from the International Collaboration on Endocarditis–Prospective Cohort Study, prosthetic valve involvement appears as an independent factor associated with mortality [2]. The deleterious role of an infection due to methicillin-resistant *Staphylococcus aureus* could appear more surprising since it has not yet been found in previous studies. However, in our opinion, it was not really studied. In the study reported by Murdoch, 869 patients exhibited a *S. aureus* IE but no data about sensitivity to methicillin was reported [2]. In a French study reporting data about 497 adults with Duke-Li–definite IE, 180 patients had a *S. aureus* IE [14]. Resistance to methicillin was observed in 13.6 % of *S. aureus*. Unfortunately, no data about impact of resistance to methicillin were provided. Finally, to the best of our knowledge, the study reported by Fowler et al. is one of the few studies providing prognostic data according to sensitivity to methicillin of *S. aureus* [26]. Among 1779 patients from the International Collaboration on Endocarditis–Prospective Cohort Study, the authors identified 424 patients with definite *S aureus* IE and no history of active IDU. Among them, 141 (33.3 %) were infected with methicillin resistant *S. aureus*. These patients tended to have higher mortality (29.8 vs. 23.3 %; *p* = 0.14) than those infected with a methicillin susceptible strain.

Our study has several limitations. First, all data were collected retrospectively. Second, it was a multicenter study. As a consequence of these 2 points, diagnostic methods, screening for complications and therapeutic measures were not standardized. Moreover, only one of the seven hospitals participating in the study had cardiac surgery units. It could thus be assumed that the access to cardiac surgery has not been the same for all patients, the most distant patients from surgical units being the least likely to benefit from emergency or urgent surgery. Similarly, a multidisciplinary approach could not be optimal for these later patients. Third, independent prognostic factors were identified by a stepwise logistic regression analysis. No case–control analysis was performed to evaluate the performance of identified factors. Fourth, our analysis was unable to establish a causal relationship between some therapeutic measures such as adequate antimicrobial treatment and survival. In a previous work, we demonstrated that such a treatment was an independent prognostic factor associated with survival [27]. Finally, we have only information on in-hospital mortality and long-term outcome was unknown.

In conclusion, mortality in patients admitted to ICU for left-sided IE remains high, especially in cases of endocarditis due to methicillin resistant *Staphylococcus aureus* and when organ failures occur and ICU scores are high.

## Abbreviations

AOR: adjusted odds ratios
CI: confident interval
CIED: cardiac implantable electronic device
IE: infective endocarditis
ICU: intensive care unit
MSSA: methicillin-susceptible Staphylococcus aureus
MRSA: methicillin-resistant Staphylococcus aureus
SAPS II: simplified acute physiology score II
SD: standard deviation
SOFA: sequential organ failure assessment
